# Association between poor self-reported health and unmarried status among adults: examining the hypothesis of marriage protection and marriage selection in the Indian context

**DOI:** 10.1186/s12889-022-14170-0

**Published:** 2022-09-22

**Authors:** Babul Hossain, K. S. James

**Affiliations:** International Institute for Population Sciences, Mumbai, 400088 India

**Keywords:** Marriage protection, Marriage selection, Unmarried women, SAGE, India

## Abstract

**Background:**

The link between marital status and health differences has long been a topic of debate. The substantial research on marriage and health has been conducted under two important hypotheses: marital protection and marriage selection. While the majority of evidence on the marriage-health relationship using these hypotheses comes from developed countries, there is a lack of evidence from Asia, particularly from India.

**Objectives:**

The current study examines theoretical frameworks of marriage i.e., marital protection and marriage selection in the Indian setting concurrently, bringing substantial empirical evidence to explore the link between marriage and health, considering this subject in the context of self-reported health (SRH). Secondly, this study will aid in investigating age and gender differences in marriage and health.

**Methods:**

Using the Study on Global AGEing and Adult Health (SAGE), a cohort study of individuals aged 50 years and older with a small section of individuals aged 18 to 49 for comparative reasons, the present study population was 25 years and above individuals with complete marital information. Logistic regressions were employed to explore the connection between marital status and self-reported health. In the marriage protection hypothesis, the follow-up poor SRH was the dependent variable, whereas the initial unmarried status was the independent variable. For the marriage selection effects, initial poor SRH as the independent variable and follow-up unmarried status as the dependent variable had considered.

**Results:**

Examining the marital protection hypothesis, the initial unmarried status (OR: 2.14; CI at 95%: 1.17, 3.92) was associated with the followed-up SRH transition from good to poor between 2007 and 2015 for young men, while initial unmarried status was linked with a lower likelihood of stable good SRH and a higher likelihood of stable poor SRH status across all age categories among women. Focusing on the marriage selection hypothesis, among young men, a significant association exists between the initial poor SRH and departure in marital status from married to unmarried. Young women with initial poor SRH (OR: 0.68; CI at 95%: 0.40, 1.00) had lower odds of stable married. In comparison, women with initially poor SRH, irrespective of age, were more likely to have higher odds of being stably unmarried.

**Conclusion:**

Marriage indeed protects health. There are also shreds of evidence on health-selected marital status in India. Taken together, the aspect of marital protection or marriage selection is gender and age-specific in India. The findings contribute to a more comprehensive understanding of the relationship between marriage and health, which may have significant implications for health-related public policies aimed at unmarried women.

**Supplementary Information:**

The online version contains supplementary material available at 10.1186/s12889-022-14170-0.

## Background

The relationship of differential marital status with health has been a subject of discussion for a long time [1–4]. Numerous approaches and methods have been used to evaluate the association between marital status and health, eventually developing two major schools of opinion.

One school of researchers argues that marital status affects individuals’ health status [5–7]. Farr [8], one of the pioneers in studying marital status and health debate, stated that marriage is a positive factor in lowering mortality among individuals than mortality among the unmarried, starting the hypothesis of marriage protection. The term “marriage protection effects” refers to the positive benefits of marriage on mortality and morbidity [6, 9, 10]. Marriage, it is assumed, strengthens social support, and wealth and prevents risky behaviour, leading to improved health. As a result, several studies have also reported that married people have lower mortality rates [11, 12], longer life expectancy [13, 14], fewer physical health problems [15, 16], are protected from life stresses and depression [17, 18], and shorter hospital stays, lower chance of nursing home admission as well as better quality health care use [19–21].

By contrast, other schools argue that individuals’ prerequisite health level explains the lower mortality and better health outcomes of married individuals than in other unmarried categories [22–24]. As per this second hypothesis, the “marriage selection theory,” healthier individuals are more likely to marry, or their marital union is less likely to change. Additionally, empirical data reveals that marriage markets exhibit positive assortative mating, which is the occurrence of mating between like people at a frequency greater than random [25]. Most of these findings are from developed countries like Sweden, the USA, Serbia, and other developed countries focusing on the complex association between marital status and health [3, 23, 24, 26–29]. Despite the long-standing links between marital status and health, studies from developing countries have mostly avoided diving further into the intricacy of the linkages between marital status and health by studying the broad marital hypothesis. It is crucial to note that the gender element was shown to attenuate the differences in marital status and health status.

Concentrating on the gender issue, a substantial body of evidence demonstrates that marriage provides women with the same health benefits as it does men; the evidence comes mostly from gender-equal countries such as North America and Europe. However, the findings on whether health influences marriage or whether marriage influences health by gender are ambiguous. For instance, Hanson et al. [30] found significant association between marriage and health only for men and men are more likely to suffer poor health status due to unmarried status. At the same time, some of the evidence suggests that marriage is more beneficial for women [31].

SRH is worth mentioning in this context since it is an important and extensively used health indicator that has been shown to be an effective indicator of objective health measures and lifestyle-related health status [32–36]. Evidence have suggested that self-reported health can predict the mortality risk, Obesity, hypertension, and metabolism [32, 35]. Simultaneously, the relationship between self-reported health (SRH) and marital status has been thoroughly explored. Although, it has been shown that married persons have a better SRH than single, divorced, widowed, or otherwise unmarried individuals, there are also mixed finding on the association between marriage and self-reported health [37–39]. For example, Fu & Noguchi [38] in their study, found that marriage affects people’s objective health by increasing their risk of developing lifestyle disease, while in terms of the selection impact; it is found that better subjective health tends to attract middle-aged and elderly Japanese to marriage. Another study by Hu [37] reported that the difference in health status between single and married rural women is mainly explained by the marital selection, whereas the difference in health status between married and widowed rural women is explained by marital protection in China.

Unlike many Western countries, marriage is still nearly universal in many south Asian countries [40]. In south Asian countries, marriage remains the cornerstone for long-term relationships, and virtually everyone marries at some point. Unmarried individuals endure enormous societal pressure to marry, which intensifies with age [41]. On the other hand, men and women who are widowed, divorced, or separated face social and economic disgrace [7]. Furthermore, many previous studies have considered the marital status as a crucial social determinant of the health and explored different dimensions of the health in the light of the marriage protection hypothesis in particular [42–46]. However, evidence is absent from the Asian context, and significantly less is known, particular from India.

In India, where male dominance continues to exist, the culture is highly normative, patrilineal, and patriarchal [47–49]. Previous research has demonstrated that gender inequalities in marriage and health outcomes strongly persist [18, 20, 50–53]. At the same time, several studies have focused on self-reported health and marital status in India. For instance, Pandey and Jha [21], using Structural Equation Modelling (SEM), concluded that poor economic circumstances had a mediation effect on the association between widowhood and poor self-reported health in India. Perkins et al. [43] found that women widowed for an extended period were more likely to have psychological distress and poor self-rated health. Further, Sudha et al. [39] suggested that even after controlling socioeconomic and family times, unmarried, particularly widows had poorer self-reported health than married older women. Further, Lloyd-Sherlock et al. [54] compared SRH status between married and widowed individuals in SAGE countries, i.e., China, Ghana, India, the Russian Federation, and South Africa, suggesting that widowed women had higher poor SRH compared to married women. Although these previous studies have given a more comprehensive range of explanations for the poor self-reported health among unmarried individuals compared to married individuals, limited studies have tried to assess the hypothesis of marriage protection and marriage selection on SRH in India. Further, less is known about how gender and age play a role in these hypotheses.

Thus, given this broader context, this study uses the Study on Global AGEing and Adult Health (SAGE), 2006–07 with followed-up data to 2015 and addresses specific questions: 1. Is there a protective or selective relationship between marriage and health? We consider this subject in the context of SRH. 2. How do gender and age play a role in analysing such a hypothesis? This study contributes to the current body of knowledge in two ways. First, this study utilizes panel data to examine theoretical frameworks of marriage in the Indian setting concurrently, bringing substantial empirical evidence to this research area. The marital protection hypothesis is examined by estimating the influence of marriage on the change in self-reported health. The marital selection hypothesis is examined by estimating health-related selection into stable and unstable marital status. Second, this study will aid in the investigation of age and gender differences in marriage and health link.

## Material & Methods

### Data source

Study on Global AGEing and Adult Health (SAGE), a cohort study of individuals aged 50 years and older with a small section of individuals aged 18 to 49 for comparative reasons, collects data on many aspects of health and other parts of socioeconomic variables in India. The SAGE baseline sample was drawn from the World Health Survey, India, 2003, encompassing six states, including Karnataka, Maharashtra, Rajasthan, Uttar Pradesh, Assam and West Bengal. SAGE’s first wave occurred in 2006–07, and the second wave occurred in 2015. New respondents in wave two were recruited to achieve sample size objectives and account for attrition and other biases associated with longitudinal survey designs. For comparison reasons, adults aged 18–49 years were included in the target sample. SAGE Wave 1 India interviewed 11,230 individuals from 9626 households, among which 4670 respondents were aged 18–49 and 6560 were 50+ years. In SAGE Wave 1, response rate was 88 and 92% for household and individuals respectively. While in the follow-up wave (SAGE Wave 2 India) included 9116 completed interviews with 1998 respondents aged 18–49 and 7118 were above 50-plus years with response rate of 95 and 77% for household and individuals respectively.

### Study population

This objective focused on the age and gender aspect of marital status, mainly focusing on married over unmarried and health. The present study population was 25 years and above individuals with full marital information. The study primarily focused on the unmarried categories, particularly those who were never married and experiencing marital union termination, i.e., divorced, separated or widowed; thus, we combined these entire sub marital groups as unmarried. A panel dataset was prepared for fulfilling this objective, focusing on the change in the marriage status and self-reported health for this objective. Thus, after excluding the new recruits by wave two, a total sample of 4077 respondents were considered for the analysis.

### Variable description

#### Main variables

Health status was assessed by self-reported health (SRH) in wave 1 and wave 2. In the survey, individuals were asked to rate their health between 1 to 5, where 1 denoted very good, and 5 denoted very bad. Thus 1 to 3 score was coded as 0 for good, and 4 to 5 was coded as 1 for bad.

Marital status was measured as a dichotomous variable, married vs unmarried, where married was coded as 0, and unmarried was coded as 1. Considerably while marriage dissolution/disruption is detrimental, being unmarried throughout life may be even more catastrophic in health status [7]. However, the fraction of persons who had never been married was seen to be minimal, since the large majority of individuals eventually get married in India. Similarly, the proportion of divorced or separated individuals in India was low. We also found the similar pattern in SAGE survey (see Additional file 1: Table 1). Thus, in the study, never-married, divorced, separated, and widowed persons were grouped together as unmarried status.

#### Control variables

Further, based on the existing literature, we included the following covariates in the analysis. The age group was categorised as the younger group (25 to 59 years) and the older group (60 and above years) [11]. The social group was divided into SC (Scheduled Caste), ST (Scheduled Tribe) and others. The respondent’s educational level was categorised as less than primary, completed secondary and above secondary [54]. Working status was categorised as yes or no [3]. While, wealth condition was divided into a poor, middle and rich category based on the household asset index [54]. Further, the chronic health problem scale was calculated based on seven self-reported health problems, including arthritis, stroke, angina, diabetes, chronic lung disease, asthma, and depression [3].

### Statistical analysis

**Fig. 1:**
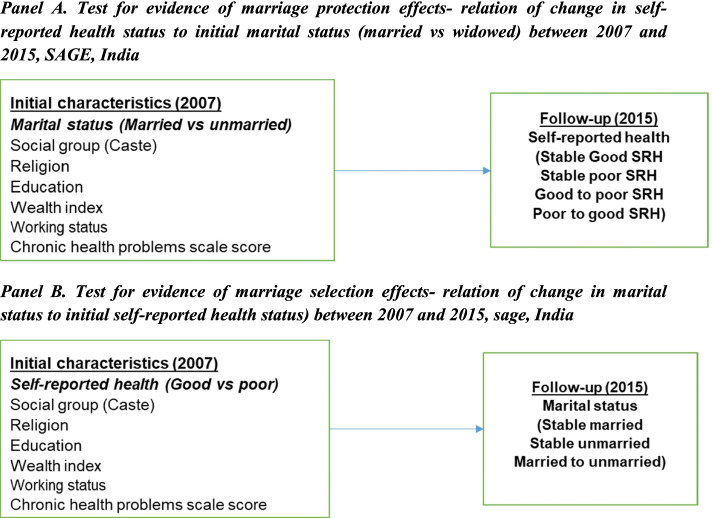
Models used in the analysis. Note: Divorced, separated, and widowed persons were grouped as nonmarried. For the test of the marriage protection hypothesis assessment, 4077 respondents were considered, while for the marriage selection assessment, 3986 respondents were considered as the sample size for unmarried to married transition between the waves were substantially insignificant

The percentage of the sample characteristics of the respondents were calculated. For the analysis, sampling weights provided by the original SAGE study were applied. Prior to the data analysis for both hypothesis tests, a logistic regression was employed to estimate the association between marital status and SRH and variables in cross-sectional analyses of waves 1 and 2 separately (see Additional file 1: Table 2 and Additional file 1: Table 3). As this study examined the hypothesis of marriage protection and marriage selection using the follow-up data, the following approaches were considered (See Fig. 1).

In the analysis, we refer to marriage protection as the process in which an individual has follow-up stable good or poor SRH or experience a transition from good to poor SRH or vice-versa due to his/her initial married or unmarried status (Fig. 1: Panel A). Logistic regressions were employed to explore the connection between initial marital status (in SAGE 1, 2007) which was considered as independent variable and follow-up self-reported health changes (in SAGE 2, 2015) as dependent variable to examine the marriage protection hypothesis [3]. The follow-up SRH was thus categorised as stable good SRH, stable poor SRH, good to poor SRH and poor to good SRH. The analysis for the marriage protection was carried out for 4077 individuals.

In the analysis, we refer to marriage selection as the process in which the individual’s follow-up marital status (stably married, stably unmarried or a departure from married to unmarried) is influenced by the his/her initial good or poor SRH (Fig. 1: Panel B). Logistic regressions were applied to investigate the association between initial SRH (in SAGE 1, 2007) and follow-up marital status (in SAGE 2, 2015) change to examine the evidence of marriage selection effects. The change in marital status from unmarried to married was not considered in the model as the number of unmarried in wave 1 became married in wave 2 was 91 and further by the age and gender stratification in each group, the sample number reduced. Thus, for marriage selection hypothesis, 3986 respondents were considered in this analysis.

The investigation took initial caste, religion, education, working status, wealth index and chronic health problem scale score as covariates in the analysis. As gender and age were two crucial factors influencing the differential marital status and its association with health outcomes, all the analyses were carried out separately for men and women in broad age groups [11]. All analysis was carried out using STATA version 15.

## Results

### Sample characteristics

Table 1 summarizes the sample characteristics of the study population aged 25 years and above in SAGE wave 1 (2007) and SAGE wave 2 (2015). Almost 15% of the samples were over the age of 60 years in 2007 which increased to 65% by the 2015. Majority of the respondents were married (approximately 90%) in 2007 and the share reduced to 82% by 2015. In contrast, almost 8% of respondents were widowed in 2007 which increased to 14% in 2015. Majority of the respondents belonged to Hindu religion and other social group. Almost two-fifth of samples lacked a primary education. 48% of the samples were poor in 2007 which reduced to 41% in 2015. 83% of the respondents were having working status in 2007 that decreased to 65% in 2015. 88% of the respondents reported poor SRH in the first wave of the SAGE survey while 83% of the respondents in second wave of the survey reported poor SRH.

**Table 1:** Study characteristics of base population, SAGE, India

Variable	SAGE Wave 1 (2007)	SAGE Wave 2 (2015)
	***N*** = 4077	
**Age**		
Below 60	85.3	36.1
60+	14.7	64.9
**Marital status**		
Never married	2.26	4.29
Currently married	89.66	81.79
Separated/divorced	0.33	0.28
Widowed	7.7	13.63
**Social group**		
SC & ST	27.8	22.7
Others	72.2	77.3
**Religion**		
Hindu	83.05	84.83
Muslim	12.82	11.86
Others	4.12	3.31
**Education**		
Less than primary	43.51	41.51
Completed secondary	34.63	36.63
Above secondary	21.86	21.86
**Wealth condition**		
Poor	48.8	41.05
Middle	19.27	19.17
Rich	31.93	39.79
**Working status**		
No	16.3	35.5
Yes	83.7	64.5
**Self-reported health (SRH)**		
Poor	87.8	82.7
Good	12.2	17.3

Source: SAGE, India

Sampling weights were applied for percentage calculation

### Change in marital status (married and unmarried) between SAGE 1 SAGE 2

**Fig. 2:**
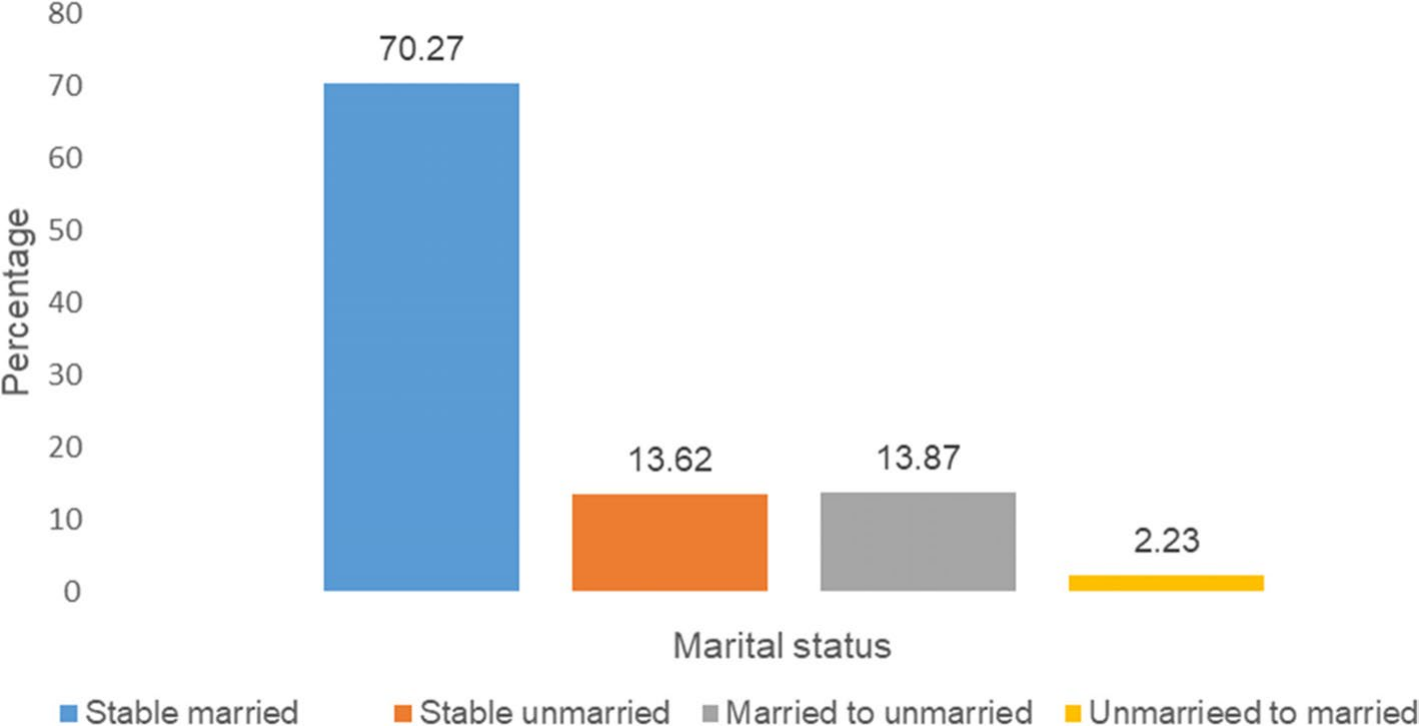
Marital status between the 2007 and 2015 among respondents aged 25 years and above, SAGE study, India (*N* = 4077)

Figure 2 illustrates the stable and unstable marital status between the two waves in SAGE survey. Between 2007 and 2015, the marital status of almost 70% of respondents as married remained unchanged. Similarly, over 13% of respondents remained unmarried between the two waves. However, we observed that between 2007 and 2015, about 14% of the samples’ marital status changed from married to unmarried. However, a small percentage of unmarried respondents in 2007 (2%) were married in 2015. This is also why we omitted respondents who were formerly unmarried and then married in a following wave from further analysis.

### Testing results on marital protection hypothesis- a panel evidence

Table 2 shows association between initial unmarried status in 2007 and follow-up self-rated health in 2015 among respondents by age and gender. Among younger men, initially unmarried status (OR: 2.1; CI at 95%: 1.17, 3.92) was significantly associated with the higher odds of reporting SRH change from good to poor between 2007 and 2015. On the contrary, the likelihood of stable and unstable poor SRH was significantly varied with initial unmarried for women. For instance, likelihood of stable good SRH between the survey points was reduced for younger (OR: 0.47; CI at 95%: 0.33, 0.67) and older (OR: 0.58; CI at 95%: 0.35, 0.94) women if the women were initially unmarried. Whereas, initially unmarried women were more likely to have stable poor SRH between the survey points irrespective of their age. The result also suggested that initially unmarried young women (OR: 2.19; CI at 95%: 1.33, 3.60), were more likely to report SRH change from good to poor between 2007 and 2015. However, we did not find any association between initial unmarried with reporting of SRH change from poor to good between 2007 and 2015 for men or women in any age group.

**Table 2:** The association between initial marital status (2007) and follow-up self-rated health (2015) among Indian respondents in the SAGE study (*N* = 4077)

	**Men**	
	25–59	60+
	﻿Odds ratio (with 95% CI)	
**﻿﻿Stable Good SRH**		
***Initial unmarried***	**0.78 (0.46, 1.34)**	**0.74 (0.51, 1.08)**
**Stable poor SRH**		
***Initial unmarried***	**0.88 (0.20, 3.86)**	**0.62 (0.28, 1.4)**
**Good to poor SRH**		
***Initial unmarried***	2.14**(1.17, 3.92)	**0.99 (0.60, 1.65)**
**Poor to good SRH**		
***Initial unmarried***	**0.51 (0.18, 1.46)**	**1.01 (0.27, 1.18)**
	**Women**	
	Odds ratio (with 95% CI)	
**Stable Good SRH**		
***Initial unmarried***	0.47***(0.33, 0.67)	0.58**(0.35, 0.94)
**Stable poor SRH**		
***Initial unmarried***	3.07***(1.47, 6.39)	3.42**(1.12, 10.46)
**Good to poor SRH**		
***Initial unmarried***	2.19***(1.33, 3.60)	**0.77 (0.42, 1.44)**
**Poor to good SRH**		
***Initial unmarried***	**1.31 (0.81, 2.13)**	**1.08 (0.54, 2.14)**

***Significance at 1%, **Significance at 5%, *Significance at 10%

Note: The stable good or stable poor SRH denotes that SRH remained the same for the individuals between two waves in the SAGE survey while Good to poor or poor to good SRH denotes the transition of SRH between two waves in the SAGE survey.

The odds represent the likelihood of stable and unstable SRH at wave 2 with compare to wave 1 of the SAGE survey, and the odds ratio is the multiplicative change in the odds for one unit of change in the given independent variable when other independent variables are controlled

### Testing results on marital selection hypothesis- a panel evidence

Table 3 demonstrates the age and gender differences in the relationship between initial poor self-rated health in 2007 and follow-up unmarried status in 2015. Between 2007 and 2015, young men (OR: 1.57; CI at 95%: 0.95, 2.58) with initially poor SRH were more likely to have a change in marital status from married to unmarried. On the other hand, young women initially having poor self-rated health (OR: 0.68; CI at 95%: 0.46, 1.00) in 2007 were less likely to experience stable married status by 2015 while young (OR: 1.72; CI at 95%: 1.13, 2.63) and old women (OR: 2.30; CI at 95%: 1.22, 4.33) with poor SRH initially were more likely to remain unmarried between the 2007 and 2015.

**Table 3:** The association between initial self-rated health (2007) and follow-up marital status (2015) among Indian respondents in the SAGE study (*n* = 3986)

	**Men**	
	25–59	60+
	Odds ratio (with 95% CI)	
**Stable married**		
***Initial poor SRH***	**0.86 (0.55, 1.35)**	**0.56 (0.41, 0.77)**
**Stable unmarried**		
***Initial poor SRH***	**0.50 (0.19, 1.33)**	**1.15 (0.67, 1.97)**
**Married to unmarried**		
***Initial poor SRH***	1.57* (0.95, 2.58)	**1.84 (1.32, 2.57)**
	**Women**	
	Odds ratio (with 95% CI)	
**Stable married**		
***Initial poor SRH***	0.68*(0.46, 1.00)	**0.47 (0.24, 0.92)**
**Stable unmarried**		
***Initial poor SRH***	1.72**(1.1, 2.63)	2.30**(1.2, 4.33)
**Married to unmarried**		
***Initial poor SRH***	**0.93 (0.54, 1.63)**	**0.57 (0.15, 2.08)**

***Significance at 1%, **Significance at 5%, *Significance at 10%

Note: The stable married or unmarried denotes that marital status remained the same for the individuals between two waves in the SAGE survey while married to unmarried status denotes the transition of marital status between two waves in the SAGE survey.

The odds represent the likelihood of stable and unstable marital status at wave 2 of the survey, and the odds ratio is the multiplicative change in the odds for one unit of change in the given independent variable, when other independent variables are controlled.

The change in marital status from unmarried to married was not considered in the model as the number of unmarried became married was only 91 and further by the age and gender stratification in each group, the sample number reduced

## Discussion

This study examines the debate on the marriage selection and marriage protection hypothesis on health, mainly focusing on self-reported health. Basic logistic regression was applied to test the marriage protection hypothesis in which follow-up SRH and initial marital status were considered. According to the findings of our study, a substantial association exist between initial unmarried status and follow-up poor SRH. Although the initial unmarried status is significantly associated with the follow-up SRH transition from good to poor between 2007 and 2015 for young men, this association is noteworthy for women. Between 2007 and 2015, we observe that initial unmarried status is strongly linked with lower likelihood of stable good SRH and higher likelihood of stable poor SRH status across all age categories among women. Between 2007 and 2015, younger unmarried women were more likely to have their SRH deteriorate from good to poor. Thus, our study demonstrates that among women, being unmarried is a risk for follow-up poor health, and the risk is stronger for women than for men.

In contrast, initial poor SRH and followed-up unmarried status have considered for testing the marriage selection hypothesis. We find that only among young men, significant association exists between the initial poor SRH with departure in marital status from married to unmarried between the two waves of survey. We also observe that only young women with initial poor SRH have lower odds of stable married and, while women irrespective of age are more likely to have higher odds of stable unmarried who had initially reported poor SRH indicating that evidence of marriage selection also exist for women in Indian context.

In line with the existing literature, the findings on the marital protection hypothesis suggest that initial marital status in wave 1 has a strong association with the followed-up poor SRH in SAGE 2, signifying that marriage has a beneficial impact on individuals [37, 38, 55–57]. Further, in our study, we find that women, particular more have health benefits through marriage [37, 38]. Thus, our findings support that the marriage protection hypothesis for health is more applicable to women, particularly younger women in India. There are various proposed pathways through which marriage may safeguard women’s health, and some of these mechanisms might explain why unmarried women are more likely to suffer from poor health than married women.

The sex-role theory may be one of the possible theories explaining our study findings on marital protection for women, particularly the young one. As per the sex-role theory, women, particularly the unemployed, are primarily dependent on their husbands for financial resources in marital unions [58–60]. At the same time, women who have experienced termination of marital union may lose a substantial amount of income and other financial resources that their spouse had given throughout the marriage or partnership. As a result, the unexpected influx of financial resources may directly or indirectly affect unmarried women’s nutritional status and living standard, influencing their objective health and other morbidity conditions, ultimately worsening self-reported health. Another possibility is that marital status is related to health-seeking behaviour, which further influences the SRH. It has been shown that married women seek more health care, obtain better quality health care, and spend more on health care than separated, divorced, or widowed women [20, 50, 61, 62]. On the other hand, evidence suggests that unmarried women neglect health-related concerns since they rely on other household members for their health-related demands, adversely influencing their perceived health state.

On the other hand, poor self-reported health is associated with lower stability of married higher stability of unmarried s across age groups among women. However, when we look at the marital selection hypothesis for men, we only find adequate evidence of a link between initial self-reported health state and follow-up marital transition from being married to being unmarried between the two waves of the survey. As a result, the marital selection concept may also apply to the Indian setting. Our evidence on the association between early health and follow-up marital status are consistent with past research [55]. Our study is in line with existing evidence. For instance, Fu and Noguchi [38] reported that poor subjective health observed to discourage middle-aged and elderly Japanese into marriage; but such impact was fairly negligible. Also, Karraker and Latham [63] found that onset of the morbidity among women can increase the risk of the marriage dissolution. While, Waldron et al. [3] found that evidence for marital selection for women who were unemployed but not for full-time workers implies that if a woman’s health problems do not interfere with her ability to work full-time, her health problems may have only minor effects on her functioning and thus have little or no effect on her marriage prospects or marriage stability. Therefore, As a result, existing evidence indicate that health issues are related to a lower chance of marriage and a higher risk of marital dissolution. Yet, in an Indian setting, employment among women is significantly low; still, our study does not find such an association as Waldron et al. [3] reported in their research. We have no explanation for the lack of evidence for marital selection effects, especially given that the impact was neither age nor gender dependent. Future researches are need to explore the marriage selection assumption in Indian setting.

### Limitations and strength

Although our study based on two waves of the SAGE survey for men and women of different ages revealed key information on marital protection and marriage selection effects, our research has substantial limitations that need to be acknowledged. Firstly, we looked at the link of initial marital status with follow-up SRH without considering potential changes in marital status in between the two waves, and similarly, we looked at the effects of initial health on marital status change without taking into account any changes in between the two waves. It will be interesting to investigate these difficulties using data sets that provide more precise information on the temporal course of changes in health and marital status. Secondly, the duration of unmarried status may have a significant impact on both health and change in unmarried status. However, because to data constraints, we were unable to account for length of unmarried status in our research [43].

## Conclusion

This research examines the marital protection and selection hypothesis in the Indian population, both broad age groups. We establish support for the marriage protection hypothesis among women using the SAGE dataset and a simple regression technique. To conclude, marriage benefits women more significantly because it provides more financial resources and benefits, enhancing perceived health. Similarly, we do find link between initial subjective health on follow-up marital status. We provide intriguing but inconclusive data in support of the marital protection and selection concept. Marriage indeed protects health. However, the aspect of marital protection is gender and age-specific in India. While, there is also evidence of marriage selection for men and women but more prominently for women. The findings contribute to a more comprehensive understanding of the relationship between marriage and health. Further, we believe that the absence of accurate longitudinal data, frequent registration of marital status and related information on health issues are significant barrier to the development of health policy and planning for vulnerable population in India. Therefore, India must enhance its civil registration systems and health information management system to ensure consistent data on marital status and health in order to develop health-related public policies that prioritize unmarried population particularly women.

## Supplementary Information

**Additional file 1: Table 1**. Marital status changed between 2007 and 2015 among adults aged 25 years and above, SAGE, India. **Table 2**. The association of marital status and other covariates with self-rated health among Indian respondents in wave 1 and wave 2, SAGE study (*N* = 4077). **Table 3**. The association between self-rated health and marital status among Indian respondents in wave 1 and wave 2, SAGE study (*N* = 4077).

## Data Availability

Data has been taken from the WHO Study on global AGEing and adult health (SAGE) [https://iipsindia.ac.in/content/SAGE-data].

## Abbreviations

SRH: Self-reported health
SAGE: Study on Global AGEing and Adult Health
OR: Odds ratio
CI: Confidence interval
