# Supplementary material for: Association between poor self-reported health and unmarried status among adults: examining the hypothesis of marriage protection and marriage selection in the Indian context
Source: BMC Public Health. 2022 Sep 22;22:1797. doi: 10.1186/s12889-022-14170-0 (PMC9494833; doi:10.1186/s12889-022-14170-0)
Supplement: Supplementary file 1 — Additional file 1: Table 1. Marital status changed between 2007 and 2015 among adults aged 25 years and above, SAGE, India. Table 2. The association of marital status and other covariates with self-rated health among Indian respondents in wave 1 and wave 2, SAGE study (N = 4077). Table 3. The association between self-rated health and marital status among Indian respondents in wave 1 and wave 2, SAGE study (N = 4077). [file 12889_2022_14170_MOESM1_ESM.docx]

| **Additional file table 1: Marital status changed between 2007 and 2015 among adults aged 25 years and above, SAGE, India** | | | | | | |
| --- | --- | --- | --- | --- | --- | --- |
|  |  | 2015 | | | | |
| 2007 |  | Never Married | Currently married | Separated/  divorced | Widowed | Total |
| Never married | No. of observation | 21 | 21 | 0 | 5 | 47 |
|  | Percentage | 44.68 | 44.68 | 0 | 10.64 | 100 |
| Currently married | No. of observation | 132 | 2,863 | 4 | 429 | 3,428 |
|  | Percentage | 3.85 | 83.52 | 0.12 | 12.51 | 100 |
| Separated/divorced | No. of observation | 0 | 3 | 13 | 9 | 25 |
|  | Percentage | 0 | 12 | 52 | 36 | 100 |
| Widowed | No. of observation | 11 | 67 | 5 | 491 | 574 |
|  | Percentage | 1.92 | 11.67 | 0.87 | 85.54 | 100 |
| Total | No. of observation | 164 | 2,954 | 22 | 934 | 4,074 |
|  | Percentage | 4.03 | 72.51 | 0.54 | 22.93 | 100 |
| Source: SAGE, India  Note: Sampling weights were applied for percentage calculation. | | | | | | |

| **Additional file table 2: The association of marital status and other covariates with self-rated health among Indian respondents in wave 1 and wave 2, SAGE study (N= 4,077)** | | | | |
| --- | --- | --- | --- | --- |
|  | **Wave 1 (2007)** | | **Wave 2 (2015)** | |
|  |  |  |  |  |
| **Men** | | | | |
|  | 25-59 | 60+ | 25-59 | 60+ |
| **Independent variables** | Odds ratio (with 95% CI) | | Odds ratio (with 95% CI) | |
| ***Unmarried*** | ***0.56 (0.23  1.36)*** | ***1.51*(0.99  2.32)*** | ***1.84 (0.88  3.83)*** | ***1.07 (0.76  1.5)*** |
| Caste | 1.02 (0.9  1.17) | 0.99 (0.88  1.11) | 1.20*(0.99  1.46) | 1.01 (0.91  1.12) |
| Religion | 1.13 (0.81  1.56) | 1.25 (0.94  1.66) | 1.08 (0.69  1.7) | 1.21 (0.95  1.53) |
| Educational level | 0.94 (0.74  1.21) | 0.86 (0.68  1.08) | 0.94 (0.68  1.29) | 0.81**(0.68  0.98) |
| Wealth status | 0.63***(0.5  0.78) | 0.64***(0.53  0.77) | 0.71**(0.54  0.95) | 0.75***(0.64  0.88) |
| Working status | 0.36***(0.24  0.54) | 0.36***(0.26  0.49) | 0.53***(0.33  0.84) | 0.42***(0.31  0.56) |
| Health score | 1.67***(1.33  2.27) | 7.42***(5.65  9.43) | 2.24***(2.01  3.56) | 9.64***(3.35  27.71) |
| **Women** | | | | |
|  | Odds ratio (with 95% CI) | | Odds ratio (with 95% CI) | |
| ***Unmarried*** | ***1.74***(1.15  2.64)*** | ***2.75***(1.48  5.10)*** | ***2.63***(1.55  4.46)*** | ***1.76***(1.24  2.5)*** |
| Caste | 0.91 (0.8  1.05) | 0.77**(0.62  0.96) | 1.06 (0.87  1.29) | 0.95 (0.84  1.09) |
| Religion | 1.29 (0.93  1.78) | 1.41 (0.75  2.66) | 1.27 (0.77  2.12) | 1.06 (0.76  1.49) |
| Educational level | 0.82 (0.58  1.16) | 0.83 (0.45  1.52) | 1.06 (0.66  1.7) | 0.61***(0.44  0.85) |
| Wealth status | 0.86 (0.68  1.09) | 0.71*(0.5  1.01) | 0.59***(0.42  0.82) | 0.82**(0.68  1) |
| Working status | 0.72 (0.47  1.08) | 0.32***(0.14  0.73) | 0.87 (0.46  1.65) | 0.32***(0.17  0.61) |
| Health score | 1.13*** (0.7  1.84) | 8.26***(7.84  9.78) | 5.49** (4.41  7.37) | 4.95*(1.13  21.64) |
| *^a^ The dependent variable in each of these logistic regressions was SRH at the cross-sectional level* | | | |  |
| *^b^ The odds represent the likelihood of having a poor SRH at different waves of the survey, and the odds ratio is the multiplicative change in the odds for one unit of change in the given independent variable when other independent variables are controlled.* | | | | |
| ****Significance at 1%, **Significance at 5%, *Significance at 10%* | | |  |  |

| **Additional file table 3: The association between self-rated health and marital status among Indian respondents in wave 1 and wave 2, SAGE study (N= 4,077)** | | | | |
| --- | --- | --- | --- | --- |
|  | **SAGE 1 (2007)** | | **SAGE 2 (2015)** | |
| **Men** | | | | |
|  | 25-59 | 60+ | 25-59 | 60+ |
| **Independent variables** | Odds ratio (with 95% CI) | | Odds ratio (with 95% CI) | |
| ***SRH*** | ***0.58 (0.24  1.39)*** | ***1.49*(0.98  2.29)*** | ***1.79 (0.86  3.75)*** | ***1.06 (0.76  1.49)*** |
| Caste | 0.83**(0.71  0.98) | 0.95 (0.82  1.09) | 0.98 (0.8  1.21) | 0.93 (0.84  1.03) |
| Religion | 0.87 (0.54  1.42) | 0.62**(0.4  0.97) | 0.63 (0.32  1.23) | 0.95 (0.73  1.23) |
| Educational level | 1.04 (0.74  1.45) | 0.84 (0.64  1.09) | 0.97 (0.67  1.42) | 1.15 (0.97  1.37) |
| Wealth status | 0.72**(0.53  0.97) | 1.03 (0.83  1.29) | 0.91 (0.65  1.26) | 0.91 (0.78  1.06) |
| Working status | 0.3***(0.18  0.51) | 0.68**(0.47  0.99) | 1.16 (0.63  2.13) | 0.62***(0.47  0.82) |
| Health score | 0.03**(0  0.92) | 0.13**(0.02  0.76) | 4.25 (0.31  58.6) | 0.07***(0.02  0.27) |
| **Women** | | | | |
|  | Odds ratio (with 95% CI) | | Odds ratio (with 95% CI) | |
| ***SRH*** | ***1.71**(1.13  2.6)*** | ***2.69***(1.46  4.98)*** | ***2.63***(1.55  4.44)*** | ***1.76***(1.24  2.51)*** |
| Caste | 1.03 (0.92  1.16) | 0.96 (0.78  1.18) | 1.02 (0.9  1.15) | 0.93 (0.83  1.04) |
| Religion | 1.17 (0.88  1.57) | 1.06 (0.59  1.91) | 0.83 (0.58  1.2) | 1.03 (0.78  1.37) |
| Educational level | 1.34**(1.03  1.74) | 1.21 (0.76  1.92) | 0.86 (0.65  1.15) | 1.19 (0.94  1.51) |
| Wealth status | 0.59***(0.48  0.72) | 0.76*(0.57  1.01) | 0.89 (0.73  1.09) | 0.92 (0.78  1.07) |
| Working status | 1.89***(1.38  2.59) | 0.76 (0.42  1.36) | 1.6**(1.08  2.37) | 0.89 (0.59  1.32) |
| Health score | 2.93 (0.44  19.58) | 0.29 (0.03  2.59) | 7.23**(1.22  42.85) | 0.5 (0.14  1.77) |
| *^a^ The dependent variable in each of these logistic regressions was marital status at cross-sectional level* | | | | |
| *^b^ The odds represent the likelihood of unmarried status at different waves of the survey, and the odds ratio is the multiplicative change in the odds for one unit of change in the given independent variable, when other independent variables are controlled.* | | | | |
| ****Significance at 1%, **Significance at 5%, *Significance at 10%* | | | |  |
